# Joint conditional Gaussian graphical models with multiple sources of genomic data

**DOI:** 10.3389/fgene.2013.00294

**Published:** 2013-12-17

**Authors:** Hyonho Chun, Min Chen, Bing Li, Hongyu Zhao

**Affiliations:** ^1^Department of Statistics, Purdue UniversityWest Lafayette, IN, USA; ^2^Department of Mathematical Sciences, University of Texas at DallasDallas, TX, USA; ^3^Department of Statistics, The Pennsylvania State University, University ParkPA, USA; ^4^Department of Biostatistics, Yale School of Public HealthNew Haven, CT, USA

**Keywords:** Gaussian graphical models, gene networks, GGMs, conditional GGMs, joint sparsity

## Abstract

It is challenging to identify meaningful gene networks because biological interactions are often condition-specific and confounded with external factors. It is necessary to integrate multiple sources of genomic data to facilitate network inference. For example, one can jointly model expression datasets measured from multiple tissues with molecular marker data in so-called genetical genomic studies. In this paper, we propose a joint conditional Gaussian graphical model (JCGGM) that aims for modeling biological processes based on multiple sources of data. This approach is able to integrate multiple sources of information by adopting conditional models combined with joint sparsity regularization. We apply our approach to a real dataset measuring gene expression in four tissues (kidney, liver, heart, and fat) from recombinant inbred rats. Our approach reveals that the liver tissue has the highest level of tissue-specific gene regulations among genes involved in *insulin responsive facilitative sugar transporter mediated glucose transport pathway*, followed by heart and fat tissues, and this finding can only be attained from our JCGGM approach.

## 1. Introduction

Inference of gene networks plays an important role in revealing the interactions among genes that may lead to a better understanding of molecular mechanisms in organisms. Biologists routinely use high-throughput technologies (e.g., microarrays) to measure gene expression data at the genome scale to study various biological and biomedical problems. Statisticians are often charged to explore interactions among genes through statistical analysis of these large data sets. It is natural to use multivariate approaches to analyze these high-throughput datasets, because multivariate methods may reveal various interactions among genes that cannot be captured from individual gene based approaches.

In this paper we focus on a graphical model approach that aims at finding relationships among a group of genes, where a graph is used for encoding relationships among multiple variables. When a graph is used for a gene network, nodes represent genes and edges represent relationships between the connected genes. The edges can be defined with various relationships among genes. For example, pairwise correlations are used to define edges in a “relevance network.” Similarly, we can define edges through conditional dependence, that is, any two genes connected with an edge in such graphical models are conditionally dependent of each other when the effects from all other genes are explained away. Therefore, when the expression profiles of two genes are correlated because they are both regulated by some other genes, the graphical model does not put an edge between these two genes because they are conditionally independent given the expressions of the common regulatory genes. In this way, the graphical model produces a more parsimonious graph than a relevance network.

Gene network inference is a complex problem, because the relationships of genes are often affected by external variables (e.g., genomic variations), and gene regulatory relationships may be altered under different conditions such as tissue types. This means that a single network inferred from gene expression measurements alone may not be adequate to describe the relationships among genes. Further, it is often desirable to jointly model gene networks under various conditions rather than considering them separately, because large parts of the networks are likely to share common topologies corresponding to similar underlying biological processes across conditions (e.g., the house keeping functions and the clock), and thus joint modeling may increase the power of detecting common gene interactions. Therefore, one may want to infer multiple condition-specific networks in a single model framework, while the network models may also need to incorporate all available external variables as well. Such inference is possible through the analysis of datasets in genetical genomic studies from same genetic origin (Jansen and Nap, 2001) where gene expressions from multiple tissues, as well as marker genotypes, are measured from the same set of individuals. These data allow us to perform an integrative analysis via joint conditional Gaussian graphical models (JCGGM) to infer relationships among genes. The JCGGM approach is an extension of the conditional Gaussian graphical model (CGGM) in order to increase power of the methods via joint modeling. The joint modeling is particularly important in the conditional models with a limited sample size, since the model's complexity increases very quickly and the separate models have no power unless appropriately combined.

**Network Inference**

- Q: What types of biological networks have been inferred in the paper?
- A: We use gene expression data and marker data from recombinant inbred rats and infer gene regulation network by using genes consisting of the insulin responsive facilitative sugar transporter mediated glucose transport pathway.
- Q: How was the quality/utility of the inferred networks assessed?
- A: Our JCGGM found that the liver network has the highest tissue specificity, and this is in line with the role of SLC2A4 protein, which forms glucose concentration gradient of muscle and fat cells, as well as the specialized glycogen breakdown of glycogen phosphorylase that only occurs in liver tissue (Watson et al., 2004; Campbell et al., 2006).
- Q: How were these networks validated?
- A: We have performed simulation study to test performance of the proposed JCGGM approach and our approach performs the best over all simulation scenarios. We have also provided the scientific literature to support the validity of the inferred networks.

In Section 2, we first introduce CGGMs and joint regularization approaches, and then propose the JCGGM that uses both the CGGM and a joint regularization approach. In Section 3, we show the performance of our approach via a simulation study and then apply it to a genetical genomics study, where gene expressions from four different tissues are measured together with genotype data from recombinant inbred rats. We show that the JCGGM approach is able to find tissue-specific gene networks. The discussion follow in Section 4.

## 2. Materials and methods

### 2.1. Material

For a real data analysis, we used a dataset of Petretto et al. (2006) in which gene expression levels in four tissues (liver, kidney, heart and fat) were measured from a panel of 29 rat recombinant inbred (RI) strains. This strain was derived from a cross between the spontaneously hypertensive rat (SHR) and the brown norway (BN) strains (Hubner et al., 2005). We downloaded the dataset normalized by the robust multi-array average (RMA) algorithm from www.genenetwork.org (Accession numbers: GN70, GN79, GN221 and GN222). From the same website, we also downloaded a genetic marker dataset that consists of 556 markers.

### 2.2. Methods

In this section, we briefly introduce recent approaches for CGGMs as well as those for joint estimation of multiple Gaussian graphical models. We then propose a new method to combine these approaches in order for inferring networks from multiple sources of biological data for finding multiple CGGMs. Finally, we explain the simulation process for generating datasets that are used for comparing the performance of our proposed method.

#### 2.2.1. A brief summary on CGGM and joint estimation of multiple GGMs

A GGM describes the conditional independences of multiple random variables, *Y*_1_, …, *Y_p_* with a graph *G* = (*V, E*), where *V* = {1, …, *p*} is a set of nodes and *E* is a set of edges, in which an edge between nodes represents that they are conditionally dependent. According to the Hammersely and Clifford theorem, a graphical model can be inferred from a factorization of the joint density of a multivariate random vector *Y* = (*Y*_1_, …, *Y_p_*)^*T*^. When *Y* is assumed to follow a multivariate Gaussian distribution *N_p_*(0, Σ), where Σ is a *p* × *p* covariance matrix, a factorization can be easily found from zero elements of the inverse covariance matrix (also known as the precision matrix), Σ^−1^ = Ω. Hence, conditional independence can be directly inferred from zero entries of a precision matrix, when a multivariate Gaussian assumption is made. This model is called a GGM (Lauritzen, 1996). Finding a sparse precision matrix with various regularizations such as lasso and adaptive lasso (Tibshirani, 1996; Zou, 2006) has been studied by many researchers including Li and Gui (2006); Yuan and Lin (2007); Friedman et al. (2008).

**Figure 1:**
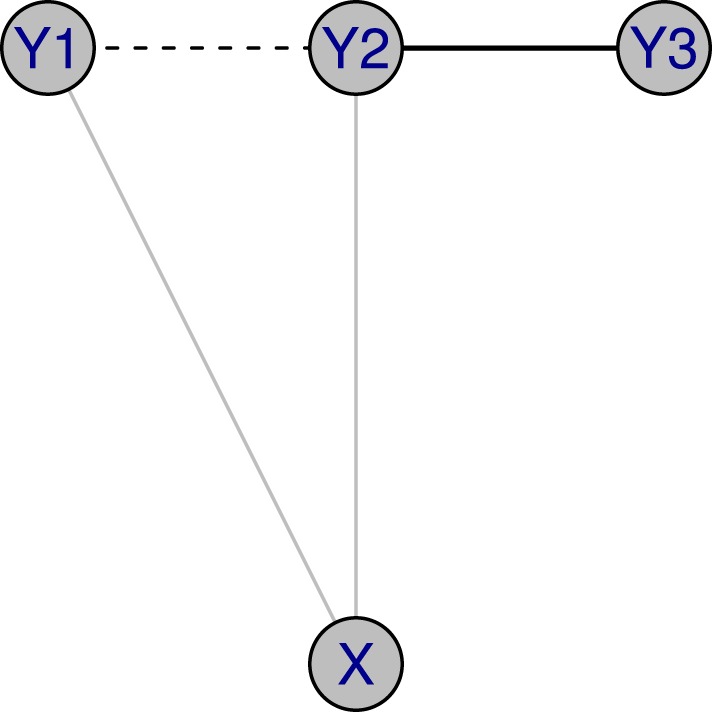
**Illustration of conditional GGM: *X* represents a single molecular marker, and *Y*_1_, *Y*_2_, *Y*_3_ represent the expressions of three genes**. When the marker effect is ignored, there are two edges in a graphical model: 1 ↔ 2 and 2 ↔ 3. After considering the marker effect, there is a single edge, represented with a solid line, in a conditional graphical model.

More recently, it has been noted that one can further elaborate a GGM by using extra sources of information. For example, as in Figure 1, let us assume that *X* represents a single molecular marker, and *Y*_1_, *Y*_2_, *Y*_3_ represent the expressions of three genes. When the marker effect is ignored, there are two edges in the unconditional graphical model: 1 ↔ 2 and 2 ↔ 3. After considering the marker effect, there is only one edge, represented by the solid line, in the conditional graphical model. For this purpose, a conditional Gaussian graphical model (CGGM) is introduced by several researchers including Yin and Li (2011); Li et al. (2012); Cai et al. (2013).

In addition to the conditional modeling, there is recently an increasing needs for inferring multiple networks that vary across conditions. For example, gene expression levels are measured in multiple tissues so as to study the tissue specificity of the gene regulations. Since the sample size is often limited, we would achieve a more accurate network inference when an appropriate joint modeling is used than when a separate estimation is made for each network because such joint analysis allows borrowing information across conditions. The joint modeling problem has been studied by several researchers including Guo et al. (2011); Danaher et al. (2013); Chun et al. (unpublished). These approaches do not accommodate the conditional models, and we will consider a joint approach in the context of estimating the conditional models.

#### 2.2.2. Joint estimation of multiple conditional gaussian graphical models

In this section, we propose an approach to estimate the multiple CGGMs jointly. This approach is aimed to infer tissue-specific gene networks from a genetical genomic dataset that consists of a marker dataset and a collection of gene expression datasets from several tissues.

We assume that at the *t*-th condition, a *p*-dimensional gene expression measurement *Y^(t)^* is from *N_p_(f^(t)^(X)*, (Ω*^(t)^*)^−1^), *t* = 1, …, *T*, where *f^(t)^*(·). is an arbitrary function, and *X* is a *q*-dimensional vector (*X*_1_, …, *X_q_*)^*T*^, describing an extra dataset such as a genetic marker dataset. We remark that *f^(t)^*(·) varies along with the condition *t*, and thus our model is able to reflect the dynamic nature of genetic controls (Gerrits et al., 2009). A conditional model describes conditional independence between any two variables, *Y_i_* and *Y_j_* given the remaining variables *Y*_−{*i, j*}_ and the extra information *f^(t)^(X)*. Here, *Y*_−{*i, j*}_ represents a *p*−2 dimensional subvector of *Y* excluding the *i* th and *j* th components. The interest is in estimating {Ω^(*t*)^}*^T^*_*t* = 1_ jointly, while accounting for the effects from *X*. We will take a two-stage approach: (1) finding consistent conditional covariance matrix ${\hat{\Sigma }}^{\left(t\right)},t=1,\ldots ,T$ and (2) finding sparse estimates of {Ω^(*t*)^}*^T^*_*t* = 1_ by using a joint sparsity penalty.

The first step is finding ${\hat{\Sigma }}^{\left(t\right)}$ with a conditional covariance matrix estimator after carefully selecting a subset of *X* that are related to *Y*. Such Σ^*(t)*^ can be estimated by using a conditional variance matrix of Σ_*YY*|*X*_, based on a conditional variance operator between RKHSs of *X* and *Y* under some general model assumptions (Li et al., 2012). Assuming the *X^(t), i^* and *Y^(t), i^*, *i* = 1, …, *n*, are independently and identically distributed random vectors as with *X^(t)^* and *Y^(t)^*, respectively, we can estimate the conditional variance matrix by using a kernel K*_X_* as follows: $\frac{1}{n}\left(Y^{\left(t\right)}{}^{T}QY^{\left(t\right)}-Y^{\left(t\right)}{}^{T}Q(QK_{X}Q){\left(QK_{X}Q\right)}^{†}QY^{\left(t\right)}\right)$, where **Y**^(**t**)^ = (*Y*^(*t*), 1^, …, *Y*^(*t*),*n*^)^*T*^, $Q=I_{n}-\frac{1}{n}J_{n}$, *I_n_* is an *n* × *n* identity matrix, *J_n_* is an *n* × *n* matrix whose elements are all 1, and *A*^†^ means a generalized inverse of a matrix *A*. When a linear kernel is used, the conditional variance matrix becomes *S_Y^(t)^Y^(t)^_* − *S_Y^(t)^X_S*^−1^_*XX*_*S_XY^(t)^_*, where $S_{XX}=\frac{1}{n}\sum_{i=1}^{n}X^{i}X^{i}{}^{T}$, $S_{XY^{\left(t\right)}}=\frac{1}{n}\sum_{i=1}^{n}X^{i}Y^{(t),i}{}^{T}$ and $S_{Y^{\left(t\right)}Y^{\left(t\right)}}=\frac{1}{n}\sum_{i=1}^{n}Y^{(t),i}Y^{(t),i}{}^{T}$. Thus, one can obtain the estimate of the conditional variance as in Yin and Li (2011); Cai et al. (2013) by using linear kernels. When *X* represents marker genotypes of a backcross from a genetical genomics study, the linear model assumption is reasonable because the genotypes have two levels of genotype values (e.g., back cross population). With other kernels such as a polynomial and a radial basis function kernel, one can model an arbitrary form of *f* flexibly.

Second, we will use a penalized profiled likelihood that jointly estimate {Ω*^(t)^}^T^*_*t* = 1_ with a joint sparsity penalization as follows:

(1) $$\begin{array}{l} \text{PPL}\left({\left\{\Omega^{\left(t\right)}\right\}}_{t=1}^{T}\right)=\sum_{t=1}^{T}n_{t}\left(-\log\text{det}\left(\Omega^{\left(t\right)}\right)+\text{tr}\left({\hat{\Sigma }}^{\left(t\right)}\Omega^{\left(t\right)}\right)\right)\\ \;+P\left({\left\{\Omega^{\left(t\right)}\right\}}_{t=1}^{T}\right), \end{array}$$

where ${\hat{\Sigma }}^{\left(t\right)}$ is the conditional covariance matrix estimate, and *P*(·) is a penalty function. In addition, *tr*(A) and *det*(A) denote trace and determinant of matrix *A*, respectively. The joint sparsity function *P*(·) can be chosen from the following different penalty functions:

- $\lambda_{1}\sum_{j\neq j^{\prime }}\sqrt{\sum_{t=1}^{T}\left|\omega_{j,j^{\prime }}^{\left(t\right)}\right|}$ (Guo et al., 2011)
- $\lambda_{1}\sum_{t=1}^{T}\sum_{j,j^{\prime }}\left|\omega_{j,j^{\prime }}^{\left(t\right)}\right|+\lambda_{2}\sum_{j,j^{\prime }}\sqrt{\sum_{t=1}^{T}\omega_{j,j^{\prime }}^{\left(t\right)}{}^{2}}$ (Danaher et al., 2013)
- $\lambda_{1}\sum_{j\neq j^{\prime }}g\left(\sum_{t=1}^{T}\left|\omega_{j,j^{\prime }}^{\left(t\right)}\right|\right)$ (Chun et al., unpublished),

where ω^*(t)*^_*j*, *j*'_ is the (*j*, *j*')th element of Ω^*(t)*^, λ_1_ and λ_2_ are positive tuning parameters, and *g* is a nonconvex function such as *g(x)* = *x*^β^, where 0 < β < 1, or a truncated log function or a truncated inverse polynomial function.

The approach of Chun et al. (unpublished) is a generalization of Guo et al. (2011), where it allows the control in balance between common and condition-specific structures by the choice of the penalty function *P*(·). Through a simulation study, Chun et al. (unpublished) showed that the truncated log penalty performs well, when the majority of networks are shared across conditions. Interestingly, the approach of Danaher et al. (2013) uses two tuning parameters, which can make the algorithm computationally challenging. Also, in their approach, the common structure is defined as $\sqrt{\sum_{t=1}^{T}\omega_{j,j^{\prime }}^{\left(t\right)}{}^{2}}$, whereas it is defined as $\sum_{t=1}^{T}\left|\omega_{j,j^{\prime }}^{\left(t\right)}\right|$ in the other approaches. With the latter choice, the condition-specific regularization can be automatically achieved by the use of a nonconvex penalty function. Additionally, they proved that the estimator from the nonconvex penalty has a sparsistency (variable selection consistency) for edges that appear in any of the conditions. We thus use the truncated log penalty of Chun et al. (unpublished) for the joint estimation of multiple GGMs. That is, our penalty function is given by

$$\begin{array}{l} P\left({\left\{\Omega \right\}}_{t=1}^{T}\right)=\sum_{j\neq j^{\prime }}\{\left(\log\left(\sum_{t=1}^{T}\left|\omega_{j,j^{\prime }}^{\left(t\right)}\right|\right)-\log\epsilon +1\right)I_{A}\\ \;+\frac{\left|\sum_{t=1}^{T}\omega_{j,j^{\prime }}^{\left(t\right)}\right|}{\epsilon }I_{A^{c}}\}, \end{array}$$

where $A=\left(\sum_{t=1}^{T}\left|\omega_{j,j^{\prime }}^{\left(t\right)}\right|>\epsilon \right)$, $A^{c}=\left(\sum_{t=1}^{T}\left|\omega_{j,j^{\prime }}^{\left(t\right)}\right|\leq \epsilon \right)$ and ϵ is a small positive constant (we used ϵ = 1*e*^−3^ in the current manuscript). We remark that the choice of a different penalty function corresponds to enforcing different level of joint sparsity in network inference. Hence we may obtain improved results from the different penalty function depending on the underlying truth. However, due to the limited sample size in biological datasets, it is often very difficult to find the optimal penalty function.

The objective function 1 can be optimized by using a local linear approximation as in Guo et al. (2011). We remark that the solution from the current optimization algorithm may not produce a global solution, and hence the choice of the good initial estimate is very important. However, our simulation study suggests that the current algorithm yields a good estimate in terms of performance of the approach. Specifically, at the (*k* + 1)th iteration, the PL is decomposed into *T* individual optimization problems as follows:

$$\begin{array}{l} {\left(\Omega^{\left(t\right)}\right)}^{\left(k+1\right)}={\text{argmin}}_{\Omega^{\left(t\right)}}n_{t}\left(tr\left(S^{\left(t\right)}\Omega^{\left(t\right)}\right)-\log\left\{\text{det}\left(\Omega^{\left(t\right)}\right)\right\}\right)\\ \;+\lambda \sum_{j\neq j^{\prime }}\zeta_{j,j^{\prime }}^{\left(k\right)}\left|\omega_{j,j^{\prime }}^{\left(t\right)}\right|, \end{array}$$

where $\zeta_{j,j^{\prime }}^{\left(k\right)}=P^{\prime }\left(\sum_{t=1}^{T}\left|{\left(\omega_{j,j^{\prime }}^{\left(t\right)}\right)}^{\left(k\right)}\right|\right)=\max{\left(\sum_{t=1}^{T}\left|{\left(\omega_{j,j^{\prime }}^{\left(t\right)}\right)}^{\left(k\right)}\right|,\epsilon \right)}^{-1}$ and (ω*^(t)^_j, j’_)^(k)^* is the solution of the previous *k*-th step. Then, the formulation becomes a single precision matrix estimation problem with a weighted lasso penalty, which can be solved by the **glasso** algorithm (Friedman et al., 2008).

**JCGGM algorithm**

1. Compute $\hat{\Sigma }$ by using a kernel. When a linear kernel is used, ${\hat{\Sigma }}^{t}=S_{Y^{t}Y^{t}}-S_{Y^{t}X}S_{XX}^{-1}S_{XY^{t}}$.
2. Initialize ${\hat{\Omega }}^{t}={\left({\hat{\Sigma }}^{t}+\delta I_{p}\right)}^{-1}$ for all 1 ≤ t ≤ T, where *I_p_* is the identity matrix and the constant δ is chosen so that ${\hat{\Sigma }}^{t}+\delta I_{p}$ is invertible. We added 1*e*^−3^ to the diagonals when the ratio of largest and smallest eigen values is larger than 1*e*^3^.
3. Update ${\hat{\Omega }}^{t}$ for all 1 ≤ t ≤ T by solving ${\text{min}}_{\Omega^{t}}tr\text{​}\left({\hat{\Sigma }}^{t}\Omega^{t}\right)-\log\left\{det\left(\Omega^{t}\right)\right\}+\lambda \text{​}\sum_{j\neq j^{\prime }}\text{​}\frac{\left|\omega_{j,j^{\prime }}^{t}\right|}{\left(\sum_{t=1}^{T}\left|{\hat{\omega }}_{j,j^{\prime }}^{t}\right|\right)},$ using a **glasso**, where ${\hat{\omega }}_{j,\;j^{\prime }}^{t}$ is the estimate from the previous step.
4. Repeat step 2 until convergence is achieved.

For selecting the tuning parameter λ, one can use the following BIC criterion:

$$\begin{array}{l} \text{BIC}(\lambda )=\sum_{t=1}^{T}\{-\log\text{det}\left({\hat{\Omega }}^{\left(t\right)}(\lambda )\right)+tr\left({\hat{\Sigma }}^{\left(t\right)}{\hat{\Omega }}^{\left(t\right)}(\lambda )\right)\\ \;+\log\left(n_{t}\right)df_{t}/n_{t}\}, \end{array}$$

where ${\left\{{\hat{\Omega }}^{\left(t\right)}(\lambda )\right\}}_{t=1}^{T}$ are the estimates from solving the penalized negative log likelihood with a tuning parameter λ where *df_t_* is card$\left\{(j,j^{\prime }):j\leq j^{\prime },{\hat{\omega }}_{j,j^{\prime }}^{\left(t\right)}\neq 0\right\}$ with *card* representing the cardinality of a finite set.

### 2.3. Methods for simulation study

For simulation study, we generate datasets by taking the number of conditions *T* = 3, the number of gene expression variables *p* = 30 and the number of markers *q* = 10. We set the sample sizes *n_t_* = 30 and 100 to assess the small and large sample performances of the estimators. We first simulate *X* that mimics a marker dataset by using *sim.map* and *sim.cross* functions from **R/qtl** package. We consider a single chromosome with length 1000 cM and place 10 equally spaced markers. We use the backcross design, since it is the design used in our real data analysis in the next section.

The scale-free network structures, which are the most commonly observed structure in biology, are generated using the Barabasi–Albert algorithm (Barabasi and Albert, 1999). We start from six edges, and add one edge at each step. We first generate common edges from each of the network structures. For each condition, randomly selected 0.1 M edges are added as condition-specific edges, where *M* is the total number of edges in the common structure. Based on the network structures, we simulate the precision matrices by setting values for the off-diagonals that correspond to edges with random numbers from *Unif*([−1,−0.5]∪[0.5,1]), and by setting the diagonal elements with ∑_*j* ≠ *i*_|ω*_i, j_*|. The process is repeated until Ω*^t^* becomes a positive definite matrix.

For simulating *Y^t^*, we first consider a scenario where there is no external variable that causes dependence among genes. This is an extreme scenario where our proposed conditional approach does not have any advantage over the unconditional model. We simulate *Y^t^* with the model *Y^t^* = *XB^t^* + *E^t^*. The elements of *B^t^* are zeros except for (1,1), (2,4) and (3,8)th positions. These nonzero coefficients are (−0.09, 0.789, −0.667), (1.361, 1.508, −2.608) and (0.687, 0.316, 2.020) for three conditions. The *i*th row of *E^t^* is simulated from *N_p_*(0, Ω*^t^*^−1^).

We then consider a scenario where there exist hotspots that cause marginal associations among genes. This is the case where our proposed conditional approach is expected to perform better than the unconditional approach. Now, *Y^t^*_1_, …, *Y^t^*_18_ are linked to *X*_1_; *Y^t^*_19_, …, *Y^t^*_25_ are to *X^t^*_4_; and *Y^t^*_26_, …, *Y^t^*_30_ are to *X*_8_. The nonzero coefficients are simulated by perturbing the coefficients used in Case 1. *B*^1^_(*i*, 1)_ = −0.09 + *N*(0, 0.1^2^), for *i* = 1, …, 18; *B*^1^_(*i*, 1)_ = 0.789 + *N*(0, 0.1^2^), for *i* = 19, …, 25; *B*^1^_(*i*, 1)_ = −0.667 + *N*(0, 0.1^2^), for *i* = 26, …, 30; *B*^2^_(*i*, 1)_ = 1.361 + *N*(0, 0.1^2^), for *i* = 1, …, 18; *B*^2^_(*i*, 1)_ = 1.508 + *N*(0, 0.1^2^), for *i* = 19, …, 25; *B*^2^_(*i*, 1)_ = −2.608 + *N*(0, 0.1^2^), for *i* = 26, …, 30;*B*^3^_(*i*, 1)_ = 0.687 + *N*(0, 0.1^2^), for *i* = 1, …, 18; and *B*^1^_(*i*, 1)_ = 0.316 + *N*(0, 0.1^2^), for *i* = 19, …, 25; *B*^1^_(*i*, 1)_ = 2.020 + *N*(0, 0.1^2^), for *i* = 26, …, 30. The *i*th row of *E^t^* is simulated from *N_p_*(0, Ω^*t*^^−1^).

## 3. Results

### 3.1. Results from simulation study

We compare the performances of unconditional/conditional GGMs and joint conditional GGMs. We use the following five criteria for the comparison:

1. False positive rate at ${\hat{\lambda }}_{\text{BIC}}$:
  $$FP({\hat{\lambda }}_{\text{BIC}})=\frac{1}{T}\sum_{t=1}^{T}\frac{\text{card}\{(i,j):i>j,\omega_{i,j}^{t}=0\text{ and }{\hat{\omega }}_{i,j}^{t}\neq 0\}}{\text{card}\{(i,j):i>j\text{ and }\omega_{i,j}=0\}}.$$
2. False negative rate at ${\hat{\lambda }}_{\text{BIC}}$:
  $$FN({\hat{\lambda }}_{\text{BIC}})=\frac{1}{T}\sum_{t=1}^{T}\frac{\text{card}\{(i,j):i>j,\omega_{i,j}^{t}\neq 0\text{ and }{\hat{\omega }}_{i,j}^{t}=0\}}{\text{card}\{(i,j):i>j\text{ and }\omega_{i,j}\neq 0\}}.$$
3. False positive rate for common zeros at ${\hat{\lambda }}_{\text{BIC}}$:
  $$\begin{array}{l} FPC({\hat{\lambda }}_{\text{BIC}})\;\\ =\frac{\begin{array}{c} \begin{array}{l} \text{card}\{(i,j):i>j;\omega_{i,j}^{t}=0\text{ for all }t=1,\ldots ,T;\text{ and }\\ \;{\hat{\omega }}_{i,j}^{t}\neq 0\text{ for any }t,1\leq t\leq T\} \end{array} \end{array}}{\text{card}\{(i,j):i>j;\text{ and }\omega_{i,j}^{t}=0\text{ for all }t=1,\ldots ,T\}}. \end{array}$$
4. False negative rate for common zeros at ${\hat{\lambda }}_{\text{BIC}}$:
  $$\begin{array}{l} FNC({\hat{\lambda }}_{\text{BIC}})\\ \;=\frac{\begin{array}{c} \begin{array}{l} \text{card}\{(i,j):i>j;\omega_{i,j}^{t}\neq 0\text{ for any }t,1\leq t\leq T;\text{ and }\\ \;{\hat{\omega }}_{i,j}^{t}=0\text{ for all }t=1,\ldots ,T\} \end{array} \end{array}}{\text{card}\{(i,j):i>j;\text{ and }\omega_{i,j}\neq 0\text{ for any }t,1\leq t\leq T\}}. \end{array}$$
5. Relative Frobenius loss (RFL):
  $$\text{RFL}=\frac{1}{T}\sum_{t=1}^{T}||\Omega^{t}-{\hat{\Omega }}^{t}|{|}_{F}^{2}/||\Omega^{t}|{|}_{F}^{2}.$$

**Figure 2:**
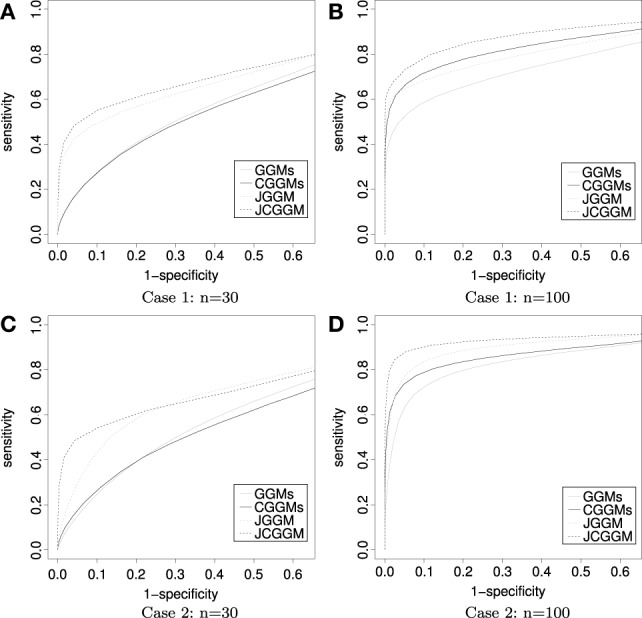
**ROC curves: the average ROC curves are presented**. Throughout all scenarios, the JCGGM performs the best. **(A)** With no external variable and a small sample size, JGGM, and JCGGM perform well. **(B)** With no external variable and a large sample size, JCGGM performs the best, followed by CGGM and JGGM. These two performs similarly. **(C)** With external variables and a small sample size, only JCGGM performs well. **(D)** With external variables and a large sample size, JCGGM performs the best, followed by JGGM and CGGM.

The results are given in Tables 1, 2. First, one can see that the joint approach improves the performance greatly for the small sample cases. This effect is more pronounced for the conditional models. This may be explained by the fact that conditional models require the estimation of more parameters than unconditional ones. Second, for large sample sizes, JCGGM performs the best in both simulation scenarios. This also confirms that even if we include extra variables in a conditional model, it will perform well as long as the sample size is large enough. The current results depend on the BIC criterion, and one may have different results when different tuning parameter selection approach is used. We thus present ROC curves in Figure 2. These ROC curves are the average ROC curves of 200 replicates. The figure confirms that JCGGM performs the best in all simulation scenarios.

**Table 1:** **Results for Case 1**.

	**FP**	**FN**	**FPC**	**FNC**	**RFL**
	***n*** = **30**				
GGMs	0.081 (0.002)	0.755 (0.004)	0.222 (0.004)	0.518 (0.008)	0.703 (0.002)
CGGMs	0.946 (0.001)	0.063 (0.002)	0.999 (0.000)	0.000 (0.000)	5087.146 (135.93)
JGGM	0.053 (0.002)	0.560 (0.004)	0.067 (0.002)	0.524 (0.005)	0.564 (0.002)
JCGGM	0.114 (0.013)	0.459 (0.007)	0.134 (0.014)	0.434 (0.008)	2.517 (0.624)
	***n*** = **100**				
GGMs	0.051 (0.001)	0.475 (0.003)	0.144 (0.003)	0.262 (0.004)	0.577 (0.001)
CGGMs	0.054 (0.001)	0.335 (0.003)	0.152 (0.003)	0.164 (0.004)	0.348 (0.002)
JGGM	0.027 (0.002)	0.383 (0.002)	0.030 (0.001)	0.346 (0.003)	0.504 (0.001)
JCGGM	0.020 (0.001)	0.329 (0.002)	0.021 (0.001)	0.298 (0.003)	0.263 (0.001)

The performances of GGMs, CGGMs, JGGMs, and JCGGMs are compared with the comparison criteria explained in subsection 3.1. When the sample size is small, the separate CGGMs select many false positives, which can be alleviated with JCGGMs. Under the scenario which is favored to JGGM, the JCGGM performs as well as the JGGM in both small and large sample cases.

**Table 2:** **Results for Case 2**.

	**FP**	**FN**	**FPC**	**FNC**	**RFL**
	***n*** = **30**				
GGMs	0.143 (0.003)	0.685 (0.005)	0.367 (0.006)	0.359 (0.007)	0.692 (0.002)
CGGMs	0.945 (0.001)	0.066 (0.002)	1.000 (0.000)	0.000 (0.000)	5343.2 (142.343)
JGGM	0.011 (0.005)	0.907 (0.006)	0.014 (0.005)	0.890 (0.006)	71.99 (71.27)
JCGGM	0.112 (0.013)	0.467 (0.008)	0.133 (0.013)	0.444 (0.008)	2.992 (0.84)
	***n*** = **100**				
GGMs	0.161 (0.002)	0.226 (0.002)	0.365 (0.004)	0.061 (0.002)	0.471 (0.002)
CGGMs	0.080 (0.001)	0.228 (0.002)	0.189 (0.003)	0.060 (0.002)	0.328 (0.002)
JGGM	0.103 (0.001)	0.164 (0.002)	0.135 (0.002)	0.132 (0.003)	0.392 (0.001)
JCGGM	0.023 (0.001)	0.162 (0.003)	0.024 (0.002)	0.127 (0.003)	0.234 (0.001)

The performances of GGMs, CGGMs, JGGMs, and JCGGMs are compared with the comparison criteria explained in subsection 3.1. When the sample size is small, the separate CGGMs select many false positives, which can be alleviated with JCGGMs. Under the scenario which is favored to JCGGMs, the JCGGM performs the best in both small and large sample cases.

### 3.2. Real data analysis

In this section, we demonstrate how to use the JCGGM approach in a real biological study. In this analysis, we focused on genes that consist of a particular pathway. Pathway information was obtained from rgd.mcw.edu, and we investigated *the insulin responsive facilitative sugar transporter mediated glucose transport pathway*. We were able to identify 34 genes in our dataset that belong to the pathway. We then used joint GGMs and joint CGGMs approach for finding a gene regulation networks. For the CGGM approach, we have selected a marker set based on **scanone** function of **R/qtl** package. For each of 34 genes, we selected markers that were significantly linked to the gene expression at the genome wide significance level of 0.05. We used permutation with 1000 replicates for computing the genome wide significance. We then took the union of those selected markers as covariates for our RKHS conditional covariance estimator with a linear kernel. We remark that the set of selected markers were tissue-specific.

**Figure 3:**
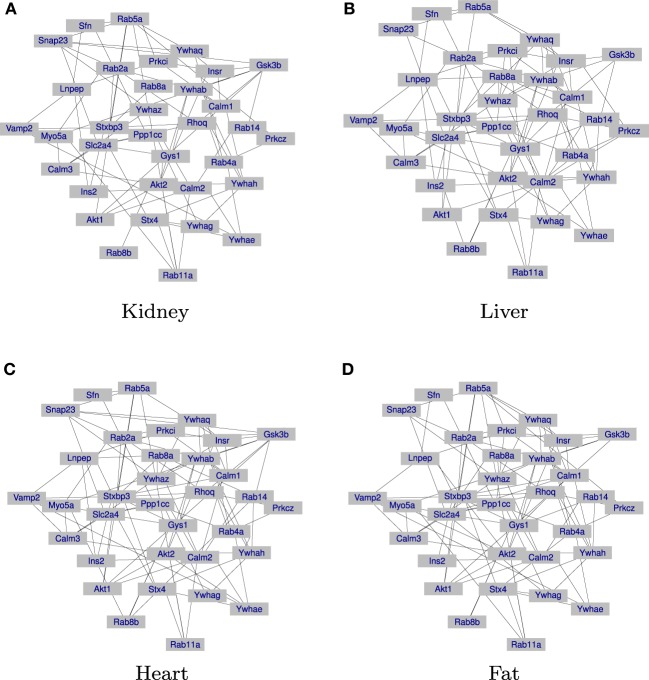
**Networks inferred from JCGGM: the liver network has the largest number of edges and the highest level of tissue-specificity**. **(A)** The inferred gene regulation network of the kidney tissue is presented. **(B)** The inferred gene regulation network of the liver tissue is presented. **(C)** The inferred gene regulation network of the heart tissue is presented. **(D)** The inferred gene regulation network of the fat tissue is presented.

The results are given in Table 3. First, in both JGGM and JCGGM, the liver networks have the largest numbers of edges. The heart and fat networks have similar numbers of edges to the liver network based on JGGM, but they have fewer edges based on JCGGM. This suggests that the pathway is the most activated in a liver tissue, and some tissue-specific controls in heart and fat might be from marker effects. We then computed the percentage of edges that present only in the corresponding tissue. Based on the JGGM, liver and heart networks have a high level of tissue-specific edges. But, the JCGGM found that the liver network has the highest tissue specificity. Interestingly, our finding is in line with the role of *SLC2A4* protein, which forms glucose concentration gradient of muscle and fat cells, as well as the specialized glycogen breakdown of glycogen phosphorylase that only occurs in liver tissue (Watson et al., 2004; Campbell et al., 2006). We also present the estimated graphs in Figure 3.

**Table 3:** **Results from JGGM and JCGGM**.

		**Kidney**	**Liver**	**Heart**	**Fat**
JGGMs	Number of edges	93	120	115	117
	% specific edges	1.1	5.8	6	4.2
JCGGMs	Number of edges	74	99	94	93
	% specific edges	0	9.1	3.2	2.1

The JGGM and JCGGM are applied to the expression measurements of genes involved in insulin responsive facilitative sugar transporter mediated glucose transport pathway. The JGGM implies that liver, heart, and fat tissues have the similar level of tissue-specificity, whereas the JCGGM implies that the liver tissue has the highest level of tissue specificity. The result from JCGGM is more convincing due to the fact that the specialized enzyme activity of glycogen phosphorylase only occurs in liver tissue.

As demonstrated in the analysis, the CGGMs can distinguish intrinsic and extrinsic regulations and gives a better overview in tissue-specificity in intrinsic regulations. To our knowledge, the tissue-specificity in gene regulations has been studied in marker-expression relationships only, and the tissue specificity in intrinsic interactions has never been studied. The JCGGMs approach can be useful for studying tissue-specificity in gene interactions.

## 4. Discussion

Genes interact with each other in various ways. Some genes interact directly, whereas some genes interact because they are both regulated by the same set of genes or other covariates. CGGM allows us to infer only direct interactions among genes by using the definition of a graphical model and using extra information as predictors. The joint sparsity regularization can be achieved by using various penalty functions. By combining these two approaches, we have explained how to find multiple CGGMs jointly and applied the approach to a real biological dataset. The analysis showed that JCGGM is able to reveal tissue-specific interactions that cannot be explained by marker effects. In addition to the previous findings on tissue specificity in gene-marker regulations, studying the extra level of tissue-specificity in gene-gene interactions brings additional understanding of the complexity in gene interactions.

In the conditional model, it is important to include all relevant extra information in the model. However, it is not necessary to include only relevant predictors, which means that one can find a better network when one incorporates available extra variables into the model as long as the sample size is large compared to the number of included variables. The RKHS approach does not involve a variable selection step of *X* because it assumes that a proper set of covariates are available. However, when the number of covariate is is large, while the sample size is small, we need to consider a variable selection step for choosing only a relevant subset of covariates. Otherwise, the RKHS conditional covariance estimator would not be consistent. The only requirement for the conditional covariance matrix estimator is that the estimator is consistent and has a finite variance [Equation 24 of Li et al. (2012)], and thus any method that can produce such an estimator can work well for finding a CGGM. For example, one can use the approaches of Yin and Li (2011) or Cai et al. (2013) as long as it yields a reasonable set of covariates. In genetical genomics study, one can use a traditional quantitative trait loci (QTL) mapping method to select relevant markers, and the eQTL mapping method was used in our manuscript.

### Conflict of interest statement

The authors declare that the research was conducted in the absence of any commercial or financial relationships that could be construed as a potential conflict of interest.
