# Supplementary material for: Joint conditional Gaussian graphical models with multiple sources of genomic data
Source: Front Genet. 2013 Dec 17;4:294. doi: 10.3389/fgene.2013.00294 (PMC3865369; doi:10.3389/fgene.2013.00294)
Supplement: Supplementary file 1 [file DataSheet1.DOC]

##function for JGGM algorithm: this takes the trainY which is a covariate adjusted responses, a nK by p matrix and trainX is a vector of length nK representing conditions.

JGGM <- function(trainX, trainY, lambda_value)

{

library(glasso)

## Set the general paramters

beta = 0 #this is for the log penalization

K <- length(unique(trainY))

p <- ncol(trainX)

diff_value <- 1e+10

count <- 0

tol_value <- 1e-2

max_iter <- 30

epsilon <- 1e-3

## Set the optimizaiton parameters

OMEGA <- array(0, c(K, p, p))

S <- array(0, c(K, p, p))

OMEGA_new <- array(0, c(K, p, p))

nk <- rep(0, K)

## Initialize Omega

for (k in seq(1, K))

{

idx <- which(trainY == k)

S[k, , ] <- cov(trainX[idx, ])

if (kappa(S[k, , ]) > 1e+3)

{

S[k, , ] <- S[k, , ] + 0.001*diag(p)

}

tmp <- solve(S[k, , ])

OMEGA[k, , ] <- tmp

nk[k] <- length(idx)

}

p2.deriv <- function(a, epsilon=1e-3){

#function for truncation

if(abs(a) < epsilon){

b <- 1/epsilon

}else{

b <- 1/abs(a)

}

return(b)

}

while((count < max_iter) & (diff_value > tol_value))

{

tmp2 <- abs(OMEGA)

tmp3 <- apply(tmp2, c(2,3), sum)

tt <- matrix(tmp3,nc=1, byrow=T)

tt2 <- apply(tt, 1, p2.deriv)

tmp <- matrix(tt2, nc=dim(tmp3)[1], byrow=T)

for (k in seq(1, K))

{

if(alpha !=1){

tt <- matrix(abs(OMEGA)[k,,],nc=1, byrow=T)

tt2 <- apply(tt, 1, p2.deriv)

tmp4 <- matrix(tt2, nc=dim(tmp3)[1], byrow=T)

V2 <- tmp*tmp4

}

penalty_matrix <- lambda_value*V2

checkeigen <-eigen(S[k,,])

if(sum(which(checkeigen$val <=0)) > 0){

S[k,,] <- S[k,,] + diag(rep(max(c(abs(checkeigen$val[which(checkeigen$val <=0)]),1e-6)),p))

}

obj_glasso <- glasso(S[k, , ], penalty_matrix, maxit=30,thr=tol_value)

OMEGA_new[k, , ] <- (obj_glasso$wi + t(obj_glasso$wi)) / 2

}

## Check the convergence

diff_value <- sum(abs(OMEGA_new - OMEGA)) / sum(abs(OMEGA))

count <- count + 1

OMEGA <- OMEGA_new

#cat(count, ', diff_value=', diff_value, '\n')

}

## Filter the noise

for (k in seq(1, K))

{

ome <- OMEGA[k, , ]

ww <- diag(ome)

ww[abs(ww) < 1e-10] <- 1e-10

ww <- diag(1/sqrt(ww))

tmp <- ww %*% ome %*% ww

ome[abs(tmp) < 0.01] <- 0

OMEGA[k, , ] <- ome

}

return(list(OMEGA=OMEGA, converg=(count == max_iter)))

}

##this is simle code for running JGGM algorithm.

### Users must input residuals from conditional models and the conditions for inputs as well as the regularization parameter.

K =3

n=100

p =10

condition <- rep(c(1:K), each=n) #condition

RY <- matrix(rnorm(3*100*p), nc= p ) ## assume that this is the residuals from QTL mapping.

fit <- JGGM(trainY=condition, trainX=RY,lambda_value=1e-3)

##solution

fit$OMEGA
